# Fertility sparing management of uterine adenosarcoma: Case report and literature review

**Published:** 2021-01-08

**Authors:** B Zizolfi, V Foreste, A Di Spiezio Sardo, A Manzi, G Bifulco, J Carugno

**Affiliations:** Department of Neuroscience Reproductive Sciences and Dentistry School of Medicine, University of Naples Federico II Naples, Italy; Department of Public Health University of Naples Federico II Naples, Italy; Obstetrics, Gynecology and Reproductive Sciences Department. Minimally Invasive Gynecology Division. University of Miami. Miller School of Medicine. Miami, FL USA

**Keywords:** Hysteroscopy, Adenosarcoma, Uterus, Fertility preservation

## Abstract

Adenosarcoma is an extremely rare malignancy of the female genital tract composed of stromal sarcoma with a benign epithelial component. Current treatment recommendations include total hysterectomy with bilateral salpingo-oophorectomy, precluding future fertility. Although most frequently diagnosed in postmenopausal women, it is occasionally present in younger women of reproductive age with desire for future fertility. In 2015, we reported the case of a 23-year-old patient diagnosed with uterine adenosarcoma, who having strong desire of future fertility, opted for fertility sparing surgery. At a follow-up five years later, we can now report her case of spontaneous pregnancy and livebirth. A review of the literature concerning fertility outcomes in patients with uterine adenosarcoma undergoing fertility sparing therapeutic options is presented.

## Introduction

Uterine adenosarcoma (UA) is a rare gynaecological malignancy composed of sarcomatous stroma and a benign epithelial component (Ulrich & Denschlag, 2018). While the epithelial component most often consists of endometrium-like tissue, the sarcomatous part is usually low-grade homologous uterine sarcoma (Pinto & Howitt, 2016). According to the extension of the sarcomatous portion of the tumour, it is defined as an adenosarcoma without sarcomatous overgrowth, when the sarcoma component occupies less than 25% of the total tumour volume, whereas it is defined as an adenosarcoma with sarcomatous overgrowth when the sarcomatous component represents more than 25 % of its volume (Carroll et al., 2014). Adenosarcomas stage I without sarcomatous overgrowth represent 90% of cases, having a 5-year overall survival up to 80% (Nathenson & Conley, 2018; Nathenson et al., 2016). Typically, adenosarcomas are associated with abnormal uterine bleeding, but may cause pelvic pain, vaginal discharge, and bulk symptoms related to uterine enlargement. The staging system for uterine sarcomas was revised in 2009 by the International Federation of Gynecology and Obstetrics (FIGO) when it was classified as distinct from endometrial carcinoma (Mbatani et al., 2018). Most adenosarcomas arise from the endometrium as polypoid tumours that typically fill and distend the uterine cavity. Adenosarcomas with sarcomatous overgrowth usually present as a large mass with fleshy, haemorrhagic, and necrotic surfaces (Mbatani et al., 2018). The most important prognostic factors of uterine adenosarcoma are age, sarcomatous overgrowth, and myometrial, lymphovascular and lymph node invasion (Arend et al., 2010). The treatment of choice is total hysterectomy with bilateral salpingo- oophorectomy (Mbatani et al., 2018), even if there is no convincing data as to the benefits of removal of the adnexa in terms of relapse rates and survival (Denschlag et al., 2019). The benefits of systematic pelvic and paraaortic lymphadenectomy are also unclear; however, systematic lymphadenectomy is not routinely recommended (Gadducci et al., 2008). Despite being more common in postmenopausal women, UA, especially in its early stages, is also being diagnosed in premenopausal women who desire future fertility. Since the standard of care for this malignancy results in permanent sterilisation, fertility sparing therapeutic options for young women desiring future fertility are often requested by the patients (Leitao & Chi, 2005).

Lee et al. (2017) described the feasibility of uterine preservation in patients diagnosed with early-stage uterine adenosarcoma. In their series of 31 patients, 7 nulliparous women were treated with uterine preservation. At 32 months follow up, 3 showed no evidence of disease, 2 had persistent disease and 2 were alive after recurrence.

A proposed fertility sparing treatment protocol includes fertility sparing surgery (FSS) consisting of a complete resection of the lesion followed by medical treatment with megestrol acetate 160 mg/day for at least 6 months. Once the desire for fertility is fulfilled, hysterectomy with bilateral salpingo-oophorectomy is recommended. A fertility sparing treatment protocol must be performed only in low-risk cases (without sarcomatous overgrowth and absent signs of metastasis or local infiltration) and close longterm follow-up is needed (Lee et al., 2017). Patients should be informed that the data on fertility-sparing therapeutic options for the treatment of adenosarcoma is limited, experimental, and is not considered standard care. We present a case of a young woman diagnosed with UA who underwent fertility sparing surgery in our institution and subsequently spontaneously conceived and delivered a healthy infant. A review of the literature including all the published cases of pregnancy after conservative treatment of UA at stage IA (tumour limited to endometrium/endocervix with no myometrial invasion) is presented. Informed consent was obtained to publish this case. The IRB (Institutional Review Board) was consulted and deemed the work exempt.

## Case Report

In 2015, a nulliparous 23-year-old woman referred to our institution was diagnosed with UA stage IA according to FIGO 2009 criteria. Due to her strong desire of future fertility, after extensive counselling she opted for FSS. Hysteroscopic complete resection of the tumour was performed, followed by oral megestrol acetate 160 mg/d as adjuvant therapy (Di Spiezio Sardo et al., 2016). This was followed by serial diagnostic hysteroscopy and an endometrial biopsy every 6 months and annual pelvic magnetic resonance imaging (MRI) from her diagnosis until 2018. In May 2018, due to her desire to conceive, the oral megestrol acetate therapy was discontinued, and she was started on cyclic vaginal progesterone. After six months without spontaneous conception, she underwent one cycle of in vitro fertilisation (IVF), without success. Surprisingly, one month after the failed assisted fertility cycle, in March 2019, she spontaneously conceived a singleton pregnancy. The patient had an uneventful antenatal course and in December 2019 had a spontaneous vaginal delivery of a full-term infant without complications. After delivery, she resumed megestrol acetate and serial hysteroscopy, endometrial biopsy and imaging follow-up protocol at the cancer centre.

## Review of literature & Discussion

Mullerian adenosarcoma is a rare gynaecological cancer composed of a benign epithelial component with sarcomatous stroma (Mbatani et al., 2018). Total hysterectomy with bilateral salpingo- oophorectomy is considered the indicated treatment of this uncommon condition, however fertility preservation is often desired when diagnosed in women of reproductive age. To the best of our knowledge, there are only four cases published in the literature of patients diagnosed with UA treated with FSS who subsequently conceived (Table 1). The first was reported by Lee et al. (2017). The authors presented a case series aiming to describe the safety and feasibility of uterine preservation in patients with UA, reporting seven patients with Stage 1 UA treated with FSS. Of the seven patients, only a 33 year-old woman was able to conceive and had a successful vaginal delivery, and remained disease free for 77 months (Lee et al., 2017). In 2018, Goh et al. (2018), reported a case of a 21 years old patient diagnosed with clinical stage 1 low-grade UA, who subsequently spontaneously conceived and had an uneventful pregnancy and full term vaginal delivery. Recently, L’Heveder et al. (2019) reported the case of a successful pregnancy and delivery of a patient diagnosed with UA treated with FSS. The authors described a case of an 18 year-old patient, who after 11 years from the FSS, underwent IVF resulting in a miscarriage at 16 weeks of gestation, likely due to cervical incompetence. She subsequently had a second IVF attempt, in which she conceived a twin gestation. Her pregnancy was complicated with a spontaneous preterm vaginal birth at 28 weeks of gestation. Thus, our case is the fourth case described in literature of pregnancy after FSS to treat UA stage 1A. As previously described (Goh et al., 2018; L’Heveder et al., 2019; Lee et al., 2017), FSS may be a feasible treatment option in case of early stage UA in which the tumour is entirely resected and does not have sarcomatous overgrowth (Table 1). It is important to highlight that currently, considering the few cases reported in literature, there is no consensus on the best follow up monitoring strategy of patients opting for fertility preservation; however, as demonstrated in our case, hysteroscopy appears to be an acceptable way to treat and to provide follow up. Hysteroscopy allows for the complete removal of the lesion, and allows us to carefully evaluate the uterine cavity and take samples for histological examination of other suspicious areas. We recommended close follow up consisting of hysteroscopy with an endometrial biopsy every 6 months and yearly MRI. Definitive surgical treatment is always offered to the patient who has the option to defer definitive surgical intervention until her desire for future fertility is fulfilled. Hysteroscopy represents a mini-invasive, safe, effective, and easy way to diagnose and treat UA, with low complication rates, preserving the uterus for future fertility.

**Table I:** Cases of pregnancy and livebirth after FSS in patient with UA stage IA (FIGO 2009).

Authors	Age at diagnosis	Clinical presentation	Treatment	Modality of follow-up	Age at conception	Type of conception	Pregnancy outcome	State of disease at the publication
Lee et al, 2017	33	NS	HSC- Mass excosopm + MPA for 3 months	NS	34	Spontaneous	Full term vaginal delivery	NED after 77 months
Goh et al, 2018	21	Heavy vaginal bleeding, endometrial polyp	HSC- Polypectomy	Interval ultrasound	24	Spontaneous	Full term vaginal delivery	Recurrence after 8 years; TLH+BSO+bilateral PLD; NED after 43 months
L'Heveder et al, 2019	18	Heavy vaginal bleeding, endometrial polyp	HSC- Polypectomy	Biannual pelvic ultrasound, hysteroscopy+ endometrial biopsy; Annual pelvic magnetic resonance imaging. After pregnancy, annual ultrasound, hysteroscopy+ endometrial biopsy.	40	IVF	Preterm vaginal delivery	NED after 20 years; TLH at patient's request.

BSO: bilateral salpingo-oophorectomy; HSC: hysteroscopy; IVF: in vitro fertilisation; MPA: medroxy-progesterone acetate, NED: No evidence of disease; NS: not specified; PLD: pelvic lymph-node dissection; TLH: total laparoscopic hysterectomy.

## Conclusion

Adenosarcoma is an extremely rare gynaecological malignancy with only a few cases reported in patients of reproductive age. The current recommended treatment is hysterectomy with bilateral salpingo- oophorectomy, however, fertility sparing treatment options are feasible in selected patients.

In cases of focal tumours without sarcomatous overgrowth and without sign of infiltration or metastasis, a fertility sparing approach may be proposed to young women diagnosed with UA and who strongly wish to preserve their fertility. There is currently insufficient evidence regarding the ideal follow up protocol for patients diagnosed with UA who are managed with FSS.
